# Atrial natriuretic peptide and leptin interactions in healthy men

**DOI:** 10.3389/fendo.2023.1195677

**Published:** 2023-06-30

**Authors:** Martin A. Daniels, Pamela Fischer-Posovszky, Michael Boschmann, Reiner Jumpertz-von Schwartzenberg, Timo D. Müller, Leontine Sandforth, Sabine Frank-Podlech, Sonja Hülskämper, Andreas Peter, Martin Wabitsch, Jens Jordan, Andreas L. Birkenfeld

**Affiliations:** ^1^ German Center for Diabetes Research (DZD e. V.), Neuherberg, Germany; ^2^ Institute of Diabetes Research and Metabolic Disease (IDM) of the Helmholtz Center Munich, University of Tübingen, Tübingen, Germany; ^3^ Department of Internal Medicine IV, Endocrinology, Diabetology and Nephrology, University Hospital Tübingen, Tübingen, Germany; ^4^ Department of Psychiatry and Psychotherapy, Charité Universitätsmedizin, Berlin, Germany; ^5^ Department of Pediatrics and Adolescent Medicine, University Medical Center Ulm, Ulm, Germany; ^6^ Division of Pediatric Endocrinology and Diabetes, Department of Pediatrics and Adolescent Medicine, University Center Ulm, Ulm, Germany; ^7^ Experimental and Clinical Research Center, Charité Universitätsmedizin Berlin and Max Delbruck Center for Molecular Medicine, Berlin, Germany; ^8^ Berlin Institute of Health, Berlin, Germany; ^9^ Institute for Diabetes and Obesity, Helmholtz Diabetes Center, Helmholtz Zentrum München, Neuherberg, Germany; ^10^ Department for Diagnostic Laboratory Medicine, Institute for Clinical Chemistry and Pathobiochemistry, University Hospital Tübingen, Tübingen, Germany; ^11^ Institute of Aerospace Medicine, German Aerospace Center (DLR), Cologne, Germany; ^12^ Chair of Aerospace Medicine, Medical Faculty, University of Cologne, Cologne, Germany; ^13^ Department of Diabetes, Life Sciences & Medicine Cardiovascular Medicine & Sciences, Kings College London, London, United Kingdom

**Keywords:** ANP, atrial natriuretic peptide, obesity, leptin, heart failure, insulin resistance, lipolysis

## Abstract

**Introduction:**

Atrial natriuretic peptide (ANP), a hormone secreted from the heart, controls cardiovascular and renal functions including arterial blood pressure and natriuresis. ANP also exerts metabolic effects in adipose tissue, liver and skeletal muscle, and interacts with the secretion of adipokines. We tested the hypothesis that ANP lowers concentrations of the anorexigenic adipokine leptin in healthy humans *in vivo*.

**Methods:**

Human ANP or matching placebo was infused intravenously (iv) into healthy men in a controlled clinical trial.

**Results:**

Within 135 minutes of iv ANP infusion, we observed an acute decrease in plasma leptin levels compared to controls. Free fatty acids markedly increased with ANP infusion *in vivo*, indicating activated lipolysis. In human SGBS adipocytes, ANP suppressed leptin release.

**Discussion:**

The study shows that the cardiac hormone ANP reduces the levels of the anorexigenic adipokine leptin in healthy humans, providing further support for ANP as a cardiomyokine in a heart - adipose tissue axis. (registered in the German Clinical Trials Register and the WHO International Clinical Trials Registry Platform was granted under **DRKS00024559**)

## Introduction

Atrial natriuretic peptide (ANP), a hormone of the natriuretic peptide family, has a well-established role in blood pressure and volume regulation (1, 2). Moreover, others and we have shown that ANP also exerts metabolic effects, such as enhancing lipolysis, lipid oxidation and mitochondrial respiration (3). ANP mediates the browning of white adipose tissue and has been implicated in the release of adipokines, including adiponectin (4, 5). In congestive heart failure as well as upon exercise, ANP concentrations increase, while in conditions such as obesity, insulin resistance, and type 2 diabetes, ANP levels decrease (1). Leptin, released by adipocytes is an anorexigenic hormone with glucose-lowering, insulin-sensitizing, and catabolic properties (6). In obesity, leptin levels increase, while they decrease during physical exercise. A combining humoral factor capable of mediating both of these effects is unknown.

*In-vitro* and *ex-vivo* studies as well as animal models (7, 8) suggested that ANP is able to reduce leptin levels from adipose tissue. Whether or not these findings translate into humans, remains unknown. Here, we tested the hypothesis that ANP reduces leptin concentrations in a *post-hoc* analysis of a placebo controlled, clinical trial involving healthy humans. To better understand the underlying mechanisms, we also applied ANP on human immortalized SGBS adipocytes (9).

## Material and methods

### Infusion studies in humans

We obtained approval by the local ethics committee and written consent from study participants for all procedures prior to the experiment. 11 healthy men without any medications underwent an overnight fast, after which we catheterized the large antecubital veins of both arms to allow for separate infusion and sampling access. After a rest period of at least 60 minutes, human ANP was infused for 135 minutes at a maximum rate of 25 ng/kg/min as previously described (10, 11). Plasma leptin concentrations were analyzed from blood drawings at baseline and at the end of ANP or placebo iv infusion at 135 mins. Plasma leptin levels were measured using ELISA (Merck Chemicals GmbH, Darmstadt, Germany). As control, 6 age- and BMI-matched healthy men underwent the same treatment, but with a placebo infusion of isotonic saline. The infusion study and leptin measurements were conducted between 2005-2007, data were analyzed in 2020 and 2021. Results of the initial trial have been reported previously (10, 11). A registration of the analysis reported here in the German Clinical Trials Register and the WHO International Clinical Trials Registry Platform was granted under **DRKS00024559**.

### Stimulation studies in SGBS cells

We cultured Simpson-Golabi-Behmel syndrome (SGBS) cells as described previously (9). After 14 day of adipogenic differentiation, cultures were treated with 10 nM human ANP (Clinalfa-Bachem #H-2095) or vehicle (PBS) in serum-free basal medium (DMEM:F12 with 3.3 mM biotin, 1.7 mM panthotenate, and antibiotics). After 24, 48, and 72 h, we collected media supernatants and either extracted total RNA or lysed cells for protein extraction. We performed RT-qPCR analysis as described previously (12). Primer sequences are available on request. Protein lysates and media supernatants were subjected to Western blot analysis as described (12) using the following antibodies: anti-β-actin (Merck #A5441), anti-leptin (Biovendor, # RD181001220).

### Statistics

Statistics were carried out using IBM SPSS Statistics Version 25. Graphs were made using GraphPad Prism 6. For parametric assessment of baseline confounders between the groups, as well as plasma leptin levels between time points 0 and 135 min in the ANP group, we used 2-tailed student’s t-test and standard deviation. For comparison of plasma leptin levels, we calculated a generalized linear model using an identity link function under a normal distribution for the leptin delta between the two groups. To assess goodness of fit and model superiority, we used Pearson Chi-Square test and Omnibus test. Wald confidence interval and Wald Chi-square metric were used for variance analysis. To assess dose dependency of plasma leptin decrease on ANP level, we calculated the Pearson correlation using a 1-tailed test of significance. Confidence levels were set at 95% and significance was assumed at α = 0.05. mRNA expression in SGBS cells was assessed using the ΔCt method, with HPRT used as reference gene. We compared mRNA levels and protein densitometry values between the groups using two-sided t-test.

## Results

### ANP infusion lowers leptin levels in healthy men compared to placebo

**Figure 1:**
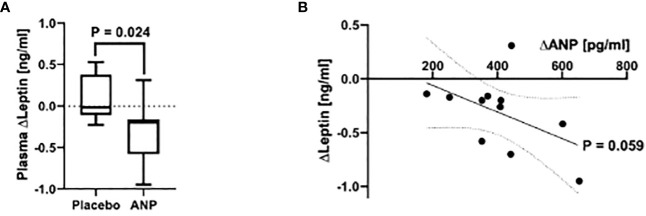
ANP reduces plasma leptin levels in healthy men. **(A)** Plasma leptin levels after acute i.v. infusion of ANP or Placebo. After infusion of ANP over 135 minutes, participants in the treatment group showed a significantly stronger decrease in plasma leptin concentrations than those in the placebo group (B = -0.201, 95% Wald CI [-0.376, -0.026] vs. B = 0.044, 95% Wald CI [-0.097, 0.185] for ANP vs. placebo, respectively; p = 0.024). **(B)** Plasma leptin levels in relation to ANP concentrations after infusion. Plasma ANP concentration showed a trend to be associated with a decrease in leptin levels in a dose-dependent manner, as shown by an inverse correlation (slope: -0.001nm/pg, Kendall’s Tau-b = -0.367, p = 0.059).

Participants in the ANP and placebo (PLC) group did not differ in age, BMI (PLC 23.0 ± 1.1 Kg/m^2^ vs ANP 24.1 ± 0.5, p=0.21) and basal leptin levels. ANP infusion over 135 minutes led to a mean increase in plasma ANP levels by 405.98 pg/ml (p < 0.001 compared to baseline, data not shown), a concentration in the high pathophysiological range observed in heart failure. ANP or PLC infusion over 135 mins did not result in any adverse events. Blood pressure did not change with PLC infusion and decreased with ANP infusion (Systolic 116 ± 3 mm Hg to 110 ± 3 mm Hg; Diastolic 63 ± 2 mmHg to 58 ± 2 mm Hg, both p<0.01) as reported previously (10, 13), in accordance with the vasodilatory and sodium excreting effect of ANP. After infusion of ANP or PLC over 135 minutes, leptin levels were lower in the ANP group compared to the PLC group (B = -0.201, 95% Wald CI [-0.376, -0.026] vs. B = 0.044, 95% Wald CI [-0.097, 0.185] for ANP vs. placebo, respectively; p = 0.024; **Figure 1A**). Assessing the ANP-treated group for dose-dependency of the relation between ANP and leptin levels, we observed a trend for an inverse correlation between ANP and leptin levels (Kendall’s Tau-b = -0.367, p = 0.059; **Figure 1B**). Simultaneously, we observed a strong and highly significant increase in plasma free fatty acid (FFA) levels in the ANP-treated group over time (300.51 ± 105.12 µmol/l at 0 min vs. 588.13 ± 198.37 µmol/l at 135 min, p < 0.001), indicative of activation of lipolysis, confirming previous work (1, 8, 10, 13).

### Treatment of a human adipocyte cell line with ANP reduced leptin secretion

**Figure 2:**
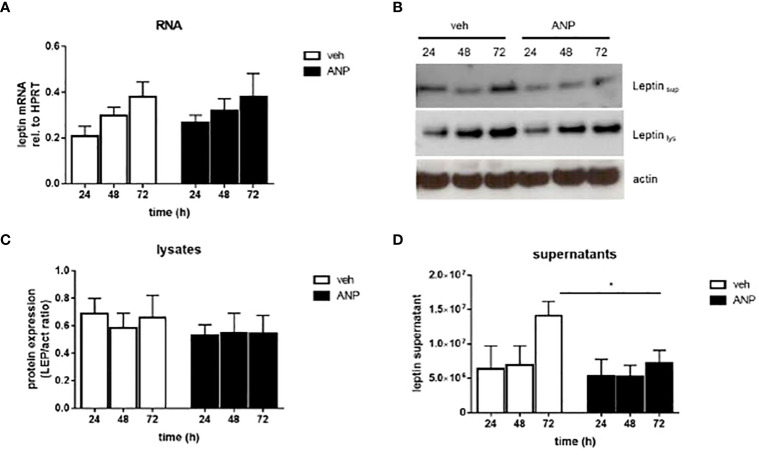
ANP inhibits leptin secretion in human adipocytes. SGBS adipocytes were treated with 10 nM ANP or vehicle in serum-free basal medium. Media supernatants, RNA and protein were harvested after 24, 48, and 72h. **(A)** The mRNA expression of leptin was assessed by qRT-PCR using the ΔCt method, HPRT was used as reference gene. Mean + SEM of 5 independently performed experiments is shown. **(B)** Leptin protein expression in cell lysates and media supernatants was determined by Western blot analysis. One representative experiment out of three performed is shown. β-actin was used as loading control. **(C, D)**. Densitometric analysis of Western blot. Mean + SEM of 3 independently performed experiments is shown. *p<0.05.

Leptin is an adipocyte-derived hormone. Therefore, we aimed to understand by which mechanism ANP reduces leptin levels. Using human SGBS adipocytes, an *in vitro* model of human adipocyte biology (9, 12), we observed no effect of ANP on leptin mRNA and protein expression (**Figures 2A-C**). However, there was a significant reduction of leptin accumulation in the media supernatants of ANP-treated vs control cells (**Figures 2B, D**). These suggest that the effect in SGBS cells was mediated by reduced leptin secretion.

## Discussion

Our results, in a *post-hoc* analysis of an interventional, placebo-controlled trial, demonstrate acute suppression of leptin levels by ANP in healthy humans. Moreover, our data suggest that the effect of ANP on leptin is dose-dependent. In the human adipocyte cell line SGBS, leptin secretion into the supernatant was lower with ANP treatment compared to vehicle treated cells, but no significant effect on leptin mRNA or intracellular lysate protein expression could be detected. These findings may indicate that ANP reduces leptin secretion, which is in line with previous *in vitro* and *ex vivo* studies (4, 5). This effect may potentially be mediated either *via* cyclic guanosine monophosphate (cGMP), the second messenger of the ANP- receptor NPR-A, or indirectly *via* free fatty acids, which have also been shown to reduce leptin secretion (14).

Free fatty acids generated during lipolysis are important intracellular signaling molecules inhibiting leptin secretion. Yet, the effect has been shown to involve leptin transcription (15), which we did not observe in SGBS cells. A more likely mechanistic explanation is a cGMP-cAMP crosstalk. While ANP signals *via* its receptor NPR-A, which is highly expressed on adipocytes, through cGMP, leptin secretion in adipocytes is reduced by increasing levels of cyclic adenosine monophosphate (cAMP). Intracellular levels of cAMP are primarily modulated by phosphodiesterase type 3B (PDE3B), which is highly expressed in adipocytes and hydrolyzes cAMP to 5’-AMP. ANP, *via* cGMP, inhibits PDE3B, ultimately increasing cAMP concentrations and thus, may reduce leptin secretion *via* this route (16).

While the reduction in leptin levels was detected within 135 minutes *in vivo*, it took longer to detect a difference in leptin secretion *in vitro*. Leptin is a 16 kD molecule that is filtered by the renal glomeruli and extensively metabolized and degraded in the renal tubules (17). We suspect that this mechanism may contribute to the different time frame in the *in vivo* and *in vitro* studies. Other mechanisms may further contribute.

ANP levels are reduced in patients with obesity (1). ANP levels increase in heart failure and with exercise (3, 18). Interestingly, leptin is increased in persons with obesity and reduced with strong physical exercise (15). While the increase in leptin concentrations in obesity is largely explained by the expansion of adipose tissue mass and leptin resistance, the reduction during exercise is less well understood. Some data suggest that the effect is mediated by improvements in leptin resistance (15), but direct mechanistic insight is lacking. ANP may be a contributing factor.

Interestingly, cross sectional studies in patients with chronic heart failure, a condition with elevated ANP and BNP concentrations, have shown increased leptin levels (19). This observation is likely explained by the fact that obesity, a condition which triggers congestive heart failure, goes along with leptin resistance and increased leptin levels. Leptin, in turn, has been hypothesized to further contribute to the progress of heart failure. From this perspective, our findings may suggest that the heart, by secreting ANP, tries to mitigate the deleterious effects of leptin on its own function. This notion is supported by observations from an inverse relationship between the natriuretic peptide BNP, which signals *via* the same receptor as ANP, and leptin in the Jackson Heart Study (20).

Hyperleptinemia and leptin resistance are a common finding in patients with obesity and insulin resistance, and a reduction in circulating leptin levels has been suggested recently to be an effective way to increase leptin sensitivity and restore its functional homeostasis in hyperleptinemic individuals, resulting in anti-obesogenic and antidiabetic effects (21). Our observation may therefore suggest that agonism of NPR-A could be an attractive therapeutic strategy to overcome leptin resistance in obesity and T2D. Interestingly, ANP overexpression and infusion in mice has shown beneficial metabolic effects, including protection from diet-induced obesity and insulin resistance. In this regard, enhancing natriuretic peptide signaling in adipose tissue, but not in muscle, protected from diet-induced obesity and insulin resistance (22). Advantages of an NPR-A agonistic compound include beneficial effects on body weight and insulin sensitivity as well as desirable cardiovascular effects, including lowering of arterial blood pressure, and preventing cardiac hypertrophy and fibrosis (1, 23, 24). Clearly, this notion warrants further research in humans.

Limitations of our analysis include the small sample size consisting of male participants, the *post-hoc* analysis and the lack of further molecular signaling mechanisms. However, the observations of our study are supported by data from cell and animal models (1, 8).

While ANP has already been implicated in a number of metabolic functions, a physiological link to leptin regulation further highlights the important role of this cardiac hormone in metabolic regulation and validates this facet of the heart - adipose axis. Given the well-established blood pressure-lowering, heart failure-protective and beneficial metabolic effects of ANP, agonists of the ANP system appear to be ideal targets for combating cardio - metabolic disease.

## Data availability statement

The original contributions presented in the study are included in the article/supplementary material. Further inquiries can be directed to the corresponding author.

## Ethics statement

The studies involving human participants were reviewed and approved by Ethics Committee of the Charite-Berlin. The patients/participants provided their written informed consent to participate in this study.

## Author contributions

AB, JJ, MB and PF-P designed the experiments. AB and PF-P performed experiments. MD, PF-P and MW analyzed data. MD, PF-P, SF-P, SH, AB wrote the manuscript. AB, SE, MB, TM, AP, MW, JJ, MD, SH, PF-P, and RJvS edited the manuscript. All authors contributed to the article and approved the submitted version.
